# Nutritional Modulation of Non-Alcoholic Fatty Liver Disease and Insulin Resistance

**DOI:** 10.3390/nu7115454

**Published:** 2015-11-05

**Authors:** Hannele Yki-Järvinen

**Affiliations:** Department of Medicine, University of Helsinki, and Minerva Foundation Institute for Medical Research, Haartmaninkatu 8, FI-00290 Helsinki, Finland; Hannele.Yki-Jarvinen@helsinki.fi; Tel.: +358-50-417-1664; Fax: +358-94-717-1896

**Keywords:** saturated fat, carbohydrate, fructose, liver fat, steatosis

## Abstract

Non-alcoholic fatty liver disease (NAFLD) covers a spectrum of disorders ranging from simple steatosis (non-alcoholic fatty liver, NAFL) to non-alcoholic steatohepatitis (NASH) and cirrhosis. NAFL increases the risk of liver fibrosis. If the liver is fatty due to causes of insulin resistance such as obesity and physical inactivity, it overproduces glucose and triglycerides leading to hyperinsulinemia and a low high-density lipoprotein (HDL) cholesterol concentration. The latter features predispose to type 2 diabetes and cardiovascular disease (CVD). Understanding the impact of nutritional modulation of liver fat content and insulin resistance is therefore of interest for prevention and treatment of NAFLD. Hypocaloric, especially low carbohydrate ketogenic diets rapidly decrease liver fat content and associated metabolic abnormalities. However, any type of caloric restriction seems effective long-term. Isocaloric diets containing 16%–23% fat and 57%–65% carbohydrate lower liver fat compared to diets with 43%–55% fat and 27%–38% carbohydrate. Diets rich in saturated (SFA) as compared to monounsaturated (MUFA) or polyunsaturated (PUFA) fatty acids appear particularly harmful as they increase both liver fat and insulin resistance. Overfeeding either saturated fat or carbohydrate increases liver fat content. Vitamin E supplementation decreases liver fat content as well as fibrosis but has no effect on features of insulin resistance.

## 1. Introduction

*NAFLD Definitions.* Non-alcoholic fatty liver disease (NAFLD) is defined as steatosis (over 5% to 10% of hepatocytes have macroscopic steatosis) [1], which is not due to excess use of alcohol (defined in the most recent guideline from American Association for the Study of Liver Diseases as alcohol consumption exceeding 21 drinks on average per week in men and 14 drinks in women) [2], or other conditions as determined by careful family and medical history, and laboratory tests to exclude at least steatosis due to viral and autoimmune causes and iron overload [2]. NAFLD is usually asymptomatic and most patients have normal transaminases (ALT <30–40 U/L for men and <20–30 U/L for women) [3,4] although NAFLD is the most common cause of incidentally discovered elevated liver function tests [5]. Some patients with NAFLD have non-alcoholic steatohepatitis (NASH), which can only be diagnosed by a liver biopsy. NASH is characterized in addition to steatosis by ballooning necrosis in the vicinity of steatotic hepatocytes, mild inflammation and possibly fibrosis [6]. Fibrosis is staged on a scale from 0 to 4, where 4 is cirrhosis [6]. While NAFLD is as common as the metabolic syndrome (MetS), the prevalence estimates of NASH range from 3% to 6% [7,8].

**Figure 1:**
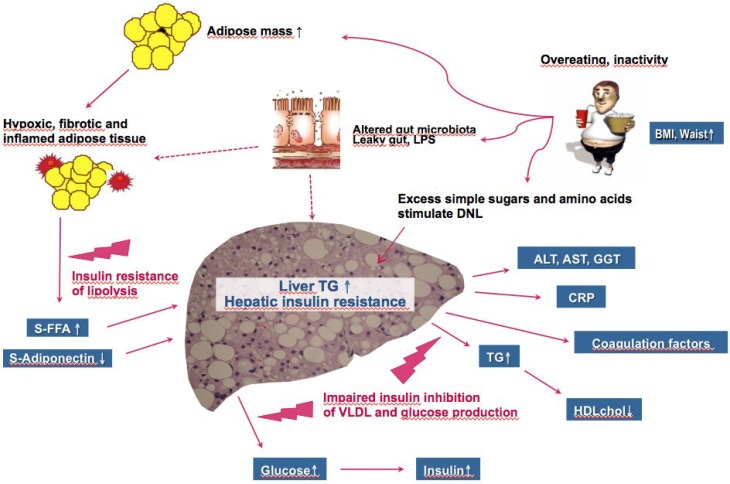
Pathophysiology of “Metabolic NAFLD”, which causes and consequences resemble those of the insulin resistance/metabolic syndrome (MetS). Overeating and physical inactivity predispose to both conditions. Excess glucose, fructose and amino acids are converted to triglyceride (TG) in the liver via de novo lipogenesis (DNL), which pathway is increased in NAFLD [17]. Alterations in gut microbiota in obesity increase gut permeability to bacterial components such as lipopolysaccharide (LPS), which may contribute to inflammation in both adipose tissue and the liver [18]. Overeating leads to adipose tissue expansion, hypoxia, increased fibrosis and cell death. Dead adipocytes are surrounded by macrophages, which produce cytokines such as tumor-necrosis alpha and chemokines such as monocyte chemoattractant protein-1. This impairs the ability of insulin to inhibit lipolysis *i.e.*, inhibit release of free fatty acids (FFA) and leads to deficiency of the insulin-sensitizing cytokine adiponectin. The latter two changes promote synthesis of intrahepatocellular TG. The ability of insulin to suppress glucose and VLDL production is impaired resulting in mild hyperglycemia and hyperinsulinemia, hypertriglyceridemia (TG**↑**) and a low HDL cholesterol concentration (HDL chol**↓**). The fatty liver also overproduces many other factors such as the liver enzymes alanine aminotransferase (ALT), aspartate aminotransferase (AST) and gamma-glutamyltransferase (GGT), C-reactive protein (CRP) and coagulation factors [13].

*Heterogeneity of NAFLD.* Although NAFLD is commonly observed in insulin-resistant obese and sometimes insulin-resistant non-obese subjects with the MetS (“Metabolic NAFLD”) (Figure 1), at least two common genetic forms of NAFLD also exist. An allele in PNPLA3 (rs738409[G], encoding I148M, prevalence 30%–50% worldwide) increases liver fat (“PNPLA3 NAFLD”) and the risk of hepatic inflammation and fibrosis, cirrhosis and hepatocellular carcinoma (HCC) [9,10,11,12]. “PNPLA3 NAFLD” is not associated with insulin resistance [10,13]. Another fairly common polymorphism, an E167K variant in (E167K) in TM6SF2 (prevalence 15%) also confers susceptibility to NAFLD (“TM6SF2 NAFLD”) [14]. Carriers with “TM6SF2 NAFLD” are at increased risk of NASH but are not insulin-resistant and their circulating concentrations of triglycerides are normal or subnormal rather than increased [14,15]. Hepatic knockdown of TM6SF2 decreases very low-density lipoprotein (VLDL) secretion [14,16].

While the PNPLA3 or TM6SF2 variants increase liver fat content and the risk of NASH, they do not increase the risk of type 2 diabetes or cardiovascular disease (CVD), consistent with lack of insulin resistance. This implies that the objective of treatment of patients with “PNPLA3 NAFLD” and TM6SF2 NAFLD’ is to prevent liver disease rather than metabolic complications. However, the same person may have “Metabolic NAFLD” and NAFLD attributable to genetic variants [15]. The etiology of NAFLD might impact the response of the liver to dietary interventions (*vide infra*).

*Significance.* Patients with NAFLD have an increased risk of mortality from CVD, cirrhosis and hepatocellular carcinoma (HCC) [19]. Recent prospective studies with paired liver biopsies contradicted the old dogma that NAFL is benign by showing that NAFL can progress to NASH and clinically significant fibrosis [8]. A recent meta-analysis of 411 patients with biopsy-proven NAFLD defined the average rate of progression. The rate of 1 stage fibrosis progression was 14.3 years for NAFL and 7.1 years for patients with NASH [20]. Given this slow rate of progression and lack of approved pharmacotherapies for NAFLD, there is abundant time for lifestyle changes to impact progression of NAFLD.

*NAFLD and insulin resistance.* The liver is the site of production of two key components of the insulin resistance/MetS, fasting serum glucose and very-low density lipoprotein (VLDL), which contains most of circulating triglycerides. In subjects with NAFLD attributable to being overweight and inactive (“Metabolic NAFLD”), the ability of insulin to normally suppress production of glucose and VLDL is impaired [21,22]. Hyperglycemia stimulates insulin secretion and thereby induces hyperinsulinemia (Figure 1). The high concentration of VLDL leads to lowering of high-density lipoprotein (HDL) cholesterol and to generation of small dense LDL particles, which are known to be highly atherogenic (see [23] for review). The liver, once fatty, also overproduces many other markers of cardiovascular risk such as C-reactive protein and coagulation factors [13] (Figure 1).

The ensuing review is focused on analyzing studies comparing effects of different diets on liver fat content and insulin sensitivity in the face of a similar total caloric content. The studies are subgrouped based on their caloric content (isocaloric/hypocaloric/hypercaloric). Studies comparing low fat/high carbohydrate to high fat/low carbohydrate diets are shown in Table 1 and studies comparing effects of different types of sugars on liver fat content and insulin sensitivity in Table 2. The influence of dietary fat on liver fat accumulation has recently been analyzed in an excellent review [24].

**Table 1:** Studies comparing effects of low fat-high carbohydrate and high fat-low carbohydrate on liver fat and insulin sensitivity.

*N*	BMI (kg/m^2^)	Age (Years)	Duration	Design	Cal	% Fat % Carb	Liver Fat (%) Before-After	Insulin Sensitivity Method Change	Year of Reference
10	33	43	2 weeks	C	ISO	16% 61%	10–8 *	fS-Ins Improved	2005 [25]
						56% 31%	10–13	Worsened	
20	29	34	3 weeks	P	ISO	20% 65%	4.0–3.5 *	Clamp NS	2011 [26]
						55% 30%	2.2–2.6	NS	
61	31	30–65	10 weeks	P	ISO	40% ^a^ 39%	3.2–2.3 *	fS-Ins NS	2012 [27]
						43% ^b^ 40%	3.2–3.5	Worsened	
45	30	35–70	8 weeks	P	ISO	28% 53%	17.7–16.1	fS-Ins NS	2012 [28]
						42% ^c^ 40%	7.4–5.2 ↓*	NS	
35	27	69	4 weeks	P	ISO	23% 57%	2.2–1.7**↓**	fS-Ins NS	2013 [29]
						43% 38%	1.2–1.6	NS	
12	32	55	6 weeks	C	ISO	21% ^d^ 49%	11.2–10.0	Clamp NS *	2013 [30]
						44% ^e^ 34%	14.2–8.6**↓** *	Improved	
22	37	44	11 weeks	P	HYPO	20% 65%	11.2–6.2 **↓**	fS-Ins Improved *	2009 [31]
						75% 10%	12.4–7.7**↓**	Improved	
18	35	45	2 weeks	P	HYPO	34% 50%	19–8.6**↓** *	fS-Ins NS	2011 [32]
						59% 8%	22–15.8**↓**	NS	
102	32	45	6 months	P	HYPO	“reduced fat”	9.6–5.6 **↓**	fS-Ins Improved	2011 [33]
						“reduced carb”	7.6–4.0**↓**	Improved	
39	23	25	7 days	P	HYPER	+fructose	12–14^h^ **↑**	fS-Ins NS	2010 [34]
						+fat	11–21^h^ **↑**	NS	
39	18–27	20–38	7 weeks	P	HYPER	40% ^f^ 43%	0.75–0.79	fS-Ins NS	2014 [35]
						36% ^g^ 48%	0.96–1.5 *	NS	

Abbreviations: N = number of completers, BMI = body mass index, yrs = years, wks = weeks, mos = months, C = crossover, P = parallel, Cal = caloric content relative to baseline diet, ISO = isocaloric, HYPO = hypocaloric, HYPER = hypercaloric, % Fat % Carb = % fat and % carbohydrate. **↑** Significant increase, **↓** significant decrease before vs. after, * significant difference in change between the two different diets, fS-Ins = fasting serum insulin, Clamp = euglycemic hyperinsulinemic insulin clamp technique. ^a^ = 10% SFA, 13% POLY; ^b^ = 20% SFA, 8% POLY; ^c^ enriched with MONO, saturated fat as in control arm; ^d^ 36% SFA/39% MONO/24% POLY; ^e^ 31% SFA/51% MONO/18% POLY; ^f^ 11% SFA and 13% POLY; ^g^ 16% SFA and 4% POLY, ^h^ Units for liver fat mmol/kg. NS = no significant change. Changes in liver fat in the table calculated based on mean changes. +fructose = addition of 3.5 grams/day of fructose/kg fat free mass, +fat = addition of 30% of total calories as fat.

**Table 2:** Effects of fructose as compared to other carbohydrates on liver fat and insulin sensitivity.

*N*	BMI (kg/m^2^)	Age (Years)	Duration	Design	Cal	Fructose Diet Other CARB Diet	Liver Fat (%) Before-After	Insulin Sensitivity Method Change	Year of Reference
32	29	34	2 weeks	P	ISO	FRU 25% ^b^	7.2–7.5	fS-Ins Worsened	2013 [36]
						GLU 25% ^b^	8.0–7.9	NS ^c^	
11	75 kg ^a^	25	7 days	C	HYPER	FRU 35% ^b^	2.1–3.2**↑**	fS-Ins NS	2010 [37]
						GLU 35% ^b^	2.1– 3.3**↑**	NS	
20	25	30	10 weeks	P	HYPER	FRU +600 cal/day	1.3–1.8	fS-Ins NS	2012 [38]
						GLU +600 cal/day	1.6–2.1	NS	
22	32	39	6 months	P	HYPER	SSB +430 cal/day	3.7–5.0↑*	fS-Ins NS	2012 [39]
						Milk +454 cal/day	12.7–11.6	NS	
64	27	42	10 weeks	P	HYPER	HFCS 8%–30% ^b^	11.8–13.7	fS-Ins NS	2013 [40]
						SUCROSE 8%–30% ^b^	14.9–13.0	NS	
32	29	34	2 weeks	P	HYPER	FRU +25% ^b^	7.2–8.9**↑**	fS-Ins NS	2013 [36]
						GLU +25% ^b^	8.0–10.1**↑**	NS	
28	22	23	6–7 days	P	HYPER	FRU 3 g/kg day	9.0–18.5**↑**	fS-Ins Worsened	2013 [41]
						GLU 3 g/kg day	12.9–16.1	NS	

Abbreviations: *N* = number of completers, BMI = body mass index, yrs = years, wks = weeks, mos = months, d = day, C = crossover, P = parallel, Cal = caloric content relative to baseline diet, FRU = fructose, HFCS = high fructose corn syrup, GLU = glucose, NS = no significant change (before *vs.* after diet), ISO = isocaloric, HYPER = hypercaloric, SSB = sugar sweetened beverage, **↑** Significant increase after vs. before, * significant difference in change between the two diets, fS-Ins = fasting serum insulin, Clamp = euglycemic hyperinsulinemic insulin clamp technique. ^a^ = body weight, ^b^ = % of total energy intake, ^c^ NS = no significant change. Changes in liver fat in the table calculated based on mean changes.

## 2. Effect of Different Diets on NAFLD and Insulin Sensitivity

**Figure 2:**
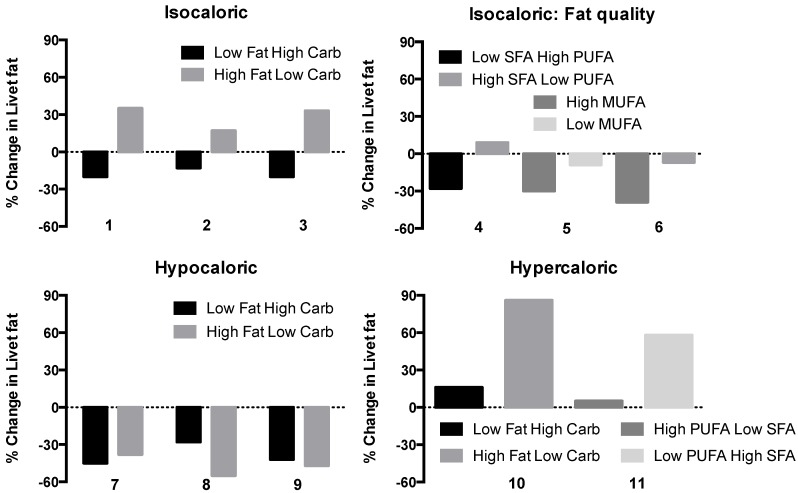
Effect of dietary composition on liver fat content, expressed as relative change from baseline measured by proton magnetic resonance spectroscopy (^1^H-MRS). Diets comparing isocaloric low fat/high carbohydrate (Low Fat High Carb) to high fat/low-carbohydrate (High Fat Low Carb) diets (upper panel on the left, 1 = [25], 2 = [26], 3 = [29]), isocaloric low saturated fat/high polyunsaturated fat (Low SFA High PUFA) to high saturated/low polyunsaturated fat (High SFA Low PUFA) or isocaloric high monounsaturated (High MUFA) to low monounsaturated fat (Low MUFA) (upper panel on the right, 4 = [27], 5 = [28], 6 = [30]) diets. The bottom panels depict effects of hypocaloric Low Fat High Carb compared to High Fat Low Carb diets (panel on the left, 7 = [31], 8 = [32], 9 = [33]) and hypercaloric Low Fat High Carb *vs.* High Fat Low Carb (10 = [34]) and High PUFA Low SFA *vs.* Low PUFA High SFA (11 = [35]) diets on liver fat content.

Overall, as shown by Figure 2, the energy content of the diet is the most important factor influencing liver fat content, which is why weight loss is recommended to all overweight or obese patients with NAFLD [2] Given that conventional hypocaloric diets are unable to achieve persistent weight loss in morbidly obese patients, bariatric surgery is becoming increasingly important in the management of NAFLD. Weight loss following bariatric surgery induces improvements in steatosis, necroinflammation and fibrosis and insulin resistance. NASH is not a contraindication for surgery unless complicated by cirrhosis or portal hypertension (see [42,43,44,45] for reviews).

Genetic etiology of NAFLD may influence the response to a hypocaloric diet. We observed a greater reduction in liver fat in response to a six-day ketogenic diet in subjects homozygous for the PNPLA3 I148M variant as compared to non-carriers [46]. This finding was recently confirmed in a larger *post hoc* analysis of 154 patients [47]. In this study, liver fat content measured by ^1^H-MRS decreased significantly and 3-fold more in subjects with the GG as compared to CC genotype (11.3% *vs.* 3.7%).

*Isocaloric comparisons (Figure 2, Table 1).* In three of the studies shown in Table 1, a low fat (16%–23% of total calories)–high carbohydrate (57%–65%) diet was compared to a low carbohydrate (31%–38%)–high fat (43%–56%) diet. Quite consistently, liver fat content decreased during the low fat-high carbohydrate diet and increased during the high fat-low carbohydrate diet (Figure 2). The high fat rather than the low carbohydrate component in the diet is the likely cause of the increase in liver fat since low carbohydrate diets seem particularly effective in reducing liver fat (*vide infra*).

In the isocaloric comparisons of a high fat–low carbohydrate *vs.* a low fat–high carbohydrate diets, there were no convincing changes in insulin sensitivity. However, the studies were small and hepatic insulin sensitivity was not assessed directly.

*Hypocaloric comparisons (Figure 2, Table 1)*. Even small amounts of weight loss decrease liver fat content. For example, two to six days of a hypocaloric diet (−1000 cal/day) decreases liver fat by 30%–45% in the face of trivial amounts of weight loss [31,46]. Two studies have compared changes in liver fat during ketogenic low-carbohydrate and standard hypocaloric diets in the face of similar decrease in body weight. In these studies lasting two days and two weeks, the ketogenic diet decreased liver fat content more than the standard hypocaloric diet [31,32]. However, in the study of Kirk *et al* [31], repeat study of the subjects after 11 weeks no longer showed a significant difference in the decrease in liver fat content (Table 1). Patients are commonly placed on a hypocaloric diet prior to bariatric surgery to reduce liver volume. Low-carbohydrate ketogenic diets decrease liver volume more than standard hypocaloric diets, most likely because low carbohydrate diets rapidly deplete liver glycogen [48].

*Hypercaloric comparisons (Figure 2, Table 1).* Rosqvist *et al.* [35] compared a diet enriched with SFAs at the expense of PUFAs to a diet rich in PUFAs but poor in SFAs. The extra calories were served as similar looking muffins. Macronutrient composition of the diets was comparable. After seven weeks, weight gain was identical in both groups (1.6 kg) but liver fat content had increased in the high SFA-low PUFA group but not in the other group (Figure 2). In the other study including 39 subjects, seven days of high fat overfeeding (30% caloric excess from fat) increased liver fat content by 86% while fructose overfeeding only increased liver fat by 16% [34]. The difference in the increments in liver fat was not statistically significant.

## 3. Effect of Type of Fat on NAFLD and Insulin Sensitivity

*High SFA–Low PUFA vs. Low SFA–High PUFA.* In the largest of the isocaloric studies [27], macronutrient composition was maintained essentially unchanged but the quality of the fat differed. Liver fat content decreased during the diet high in PUFA and low in SFA compared to the high SFA–low PUFA diet. This suggests that either SFAs or PUFAs regulate liver content. Fasting insulin increased in the high SFA-low PUFA group and remained unchanged in the high PUFA-low SFA group (Table 1). This result supports previous data such as the KANWU study in 162 subjects showing that isocaloric substitution of dietary SFA for MUFA for three months impairs insulin sensitivity and lowers LDL cholesterol [49]. The impairment in insulin sensitivity was due to the SFA rather than the MUFA diet, consistent with several smaller studies [50].

*High MUFA.* Two isocaloric studies compared the effect of increasing total fat content using mainly MUFAs [28,30]. Although total fat content was higher both in the study of Bozzetto *et al.* (42% *vs.* 28%) [28] and the study of Ryan *et al.* (44% *vs.* 21%) [30], liver fat content decreased with the diets enriched with MUFAs. However, the lower carbohydrate content of the MUFA could also have contributed to the lowering of liver fat content.

As summarized in an extensive review, MUFAs do not impair insulin sensitivity as do SFAs [50]. For example, MUFAs did not affect insulin sensitivity In the “KANWU” study [49] or in the more recent large 24-week “RISCK” study where subjects were randomized to consume either a high SFA or a high MUFA diet [51].

## 4. Effect of Type of Carbohydrate on NAFLD and Insulin Sensitivity

In contrast to glucose, fructose bypasses the rate-limiting step of glycolysis catalyzed by phosphofructokinase in the liver (see [52] for review). It therefore can provide more substrates for de novo lipogenesis DNL than glucose and could be predicted to increase intrahepatic triglycerides and VLDL production more than glucose [52]. Five studies have compared effects of fructose or high fructose corn syrup to glucose or sucrose overfeeding on liver fat content (Table 2). Of these, only one short-term study found fructose but not glucose to increase liver fat [41]. Fructose did not induce insulin resistance in any of the studies. Maersk *et al.* found six months of sugar-sweetened beverage (SSB) to increase liver fat more than milk [39]. However, milk contains only half as much carbohydrate as SSB the rest being protein and fat. These data and the systematic review and meta-analysis by Chung *et al.* [53] imply that there is not enough evidence to draw conclusions regarding effects of fructose and HFCS compared to sucrose consumption on NAFLD. However, even if a difference in changes in liver fat between the fructose and glucose was not demonstrated, excess carbohydrate calories increased liver fat content in many of the comparative studies as has also been found in high sugar overfeeding studies without another carbohydrate comparator arm [34,46,54,55]. Although not found in studies quantifying liver fat (Table 2), isocaloric fructose (25% of total daily caloric intake for 10 weeks) as compared to glucose consumption has been shown to impair insulin sensitivity [56].

## 5. Effect of Other Nutritional Interventions on NAFLD and Insulin Sensitivity

*Vitamin E.* Vitamin E is an antioxidant which, at a dose of 800 IU/day was shown to improve steatosis, inflammation and ballooning in a 96-week trial in NASH [57]. The vitamin E arm included 84 subjects and placebo arm 83 subjects with NASH but with no diabetes. Vitamin E did not change insulin sensitivity or lipid concentrations. Vitamin E at a dose of 800 IU/day compared to placebo has also been shown to resolve NASH in children aged 8–17 years with biopsy-proven NAFLD [58].

## 6. Mechanisms Underlying Nutritional Modulation of Liver Fat Content in Humans

Fatty acids in intrahepatocellular triglycerides can originate from adipose tissue lipolysis, DNL, uptake of fatty acids from chylomicron remnants and from fatty acids released during intravascular hydrolysis of triglyceride-rich lipoproteins [59]. Studies using stable isotopes to trace pathways of hepatic triglyceride synthesis have shown that both adipose tissue lipolysis and DNL are increased in NAFLD [17,60,61,62] (Figure 1).

*Nutritional modulation of lipolysis.* Short-term fasting lowers glucose and insulin concentrations and increases the rate of basal lipolysis [46,63,64]. The increase rate of lipolysis, as measured by a stable isotope glycerol tracer is closely related to decreases in fasting insulin [63]. After an overnight fast, approximately 40% of the FFA are taken up by the splanchnic bed. These FFA can be used for ketone body production, oxidation or secreted in VLDL. Over six days of a hypocaloric low-carbohydrate diet, liver fat decreases despite an increase in lipolysis as FFA as are oxidized and used for ketone body production rather than stored as triglycerides in the liver [46]. Whether these changes were due to the decrease in energy content of the diet or the low carbohydrate content is unclear as very few data comparing effects of different diets on liver fat content and lipolysis are available. In the study of Haufe *et al.* comparing low and high carbohydrate hypocaloric diets [33], fasting FFA increased during the low carbohydrate diet but remained unchanged during the high carbohydrate diet [33]. As reviewed by Jensen *et al.*, isoenergetic diets do not seem to change basal FFA flux or oxidation [65].

*Nutritional modulation of DNL.* Short-term overfeeding with carbohydrate as compared to fat markedly increases DNL [66,67], which in relative terms is the most increased pathway in NAFLD [17]. However, in absolute terms, lipolysis is the main source of intrahepatocellular triglycerides both in normal subjects and those with NAFLD [17]. There are no studies comparing effects of different diets on liver fat content and DNL.

## 7. Concluding Remarks

Studies comparing effects of different diets on liver fat content and insulin sensitivity have included a low number of subjects and lasted a maximum of six months. Nevertheless, some conclusions seem justified. Hypocaloric diets decrease while overfeeding increases liver fat content. Low fat–high carbohydrate as compared to high fat–low carbohydrate diets seem to decrease liver fat and enhance insulin sensitivity in the face of similar isocaloric or hypocaloric total caloric contents. The deleterious effect of high fat seems to be due to SFAs while PUFA or MUFA containing diets may be beneficial. Hypercaloric high carbohydrate diets increase liver fat content, but there are no convincing data to show fructose is worse than glucose, although the metabolism of fructose can be predicted to have more harmful effects on the liver than glucose. The sources of intrahepatocellular triglycerides or impact of genetic forms of NAFLD during different diets have not been systematically studied. Given the high prevalence of both “Metabolic NAFLD”, “PNPLA3 NAFLD” and “TM6SF2 NAFLD” and the associated risks of type 2 diabetes and CVD (“Metabolic NAFLD”) and advanced liver disease (all forms increase the risk of NASH, cirrhosis and HCC), there is a need for large multicenter studies with sufficient numbers of patients to define the composition of a diet which can prevent or reverse these problems.

## Abbreviations

AASLD: American Association for the Study of Liver Diseases
ALT: alanine aminotransferase
AST: Aspartate aminotransferase
CRP: C-reactive protein
CVD: cardiovascular disease
DNL: de novo lipogenesis
GGT: gamma-glutamyltransferase
HCC: hepatocellular carcinoma
HDL: high-density lipoprotein
LDL: low-density lipoprotein
MetS: metabolic syndrome
MUFA: monounsturated fatty acid
NAFL: non-alcoholic fatty liver
NAFLD: non-alcoholic fatty liver disease
NASH: non-alcoholic steatohepatitis
PNPLA3: patatin-like phospholipase domain containing 3
PUFA: polyunsaturated fatty acid
SFA: saturated fatty acid
SSB: sugar-sweetened beverages
TM6SF2: transmembrane 6 superfamily member 2
VLDL: very low-density lipoprotein
